# Date (*Phoenix dactylifera* L.) seed oil is an agro-industrial waste with biopreservative effects and antimicrobial activity

**DOI:** 10.1038/s41598-023-44251-y

**Published:** 2023-10-10

**Authors:** Hana Alkhalidy, Anas A. Al-Nabulsi, Marah Al-Taher, Tareq Osaili, Amin N. Olaimat, Dongmin Liu

**Affiliations:** 1 Department of Nutrition and Food Technology, Faculty of Agriculture, Jordan University of Science and Technology, P.O. Box 3030, Irbid, 22110 Jordan; 2 Department of Clinical Nutrition and Dietetics, College of Health Sciences, The University of Sharjah, P.O. Box 27272, Sharjah, UAE; 3 Department of Clinical Nutrition and Dietetics, Faculty of Applied Medical Sciences, The Hashemite University, Zarqa, 13133 Jordan; 4 Department of Human Nutrition, Foods and Exercise, College of Agriculture and Life Sciences, Virginia Tech, Blacksburg, VA 24061 USA

**Keywords:** Infectious diseases, Antimicrobials

## Abstract

Antimicrobial resistant (AMR) infections are a leading health threat globally. Previous literature has underscored the farm-to-fork continuum as a potential focal point for the emergence and spread of AMR. In the present study, date (*Phoenix dactylifera* L.) seed oil was investigated for its chemical composition and antimicrobial activity against common foodborne pathogens including *Escherichia coli O157:H7, Salmonella* enteritidis*, Salmonella typhimurium*, *Listeria monocytogenes*, and *Staphylococcus aureus *in vitro, and in ultra-high-temperature (UHT) milk as a food model at storage temperatures of 37 °C (24 h) and 10 °C (7 days). GC–MS analysis of the seed oil revealed 20 compounds, with octadecane (52.2–55.4%) as the major constituent, and the fatty acid analysis revealed 17 fatty acids, with oleic acid (42.3–43.1%) as the main constituent, followed by lauric acid (19.8–20.3%). The antimicrobial activity of date seed oil was determined using the microdilution method. A significant inhibition against gram-negative bacteria was noted in microbiological media and UHT milk, with a log reduction ranging from 4.3 to 6.7 (at 37 °C/24 h) and 5.7 to 7.2 (at 10 °C/7 days), respectively, at oil concentrations ranging between 10 and 15 µl/ml. The oil showed a similar significant inhibitory effect against *St. aureus* in the microbiological media (2.0–6.0 log reduction), whereas the inhibitory effect against *L. monocytogenes* was not statistically significant, with a maximum log reduction of 0.64 achieved at a concentration of 10 µl/ml. AFM imaging of the bacteria showed that oil treatment led to morphological changes in the bacteria including the formation of distorted shapes, surface blebs, indentations, stiffness, and swelling. Present findings suggest that date seed oil can be a promising by-product with potential antimicrobial activity and a food preservative.

## Introduction

In food manufacturing and packaging industries, antimicrobials play a pivotal role in ensuring the safety of perishable food products such as dairy and meat products^1^. These products require protection from the growth of spoilage and pathogenic bacteria throughout the different stages of preparation, storage, and distribution to achieve the desired shelf life^2^. And such foods are consistently susceptible to acquiring and harboring foodborne pathogens from the farm to the consumer’s table^1,2^. However, in response to the imperative of seeking alternative strategies to combat the global challenge of antimicrobial resistance (AMR)^1,3^ and driven by the evident shift in consumer preferences towards food production systems that are both health-conscious and environmentally sustainable, a noteworthy trend has emerged. This trend has catalyzed the exploration of innovative biopreservation strategies that pivot towards the utilization of natural antimicrobial agents as opposed to synthetic preservatives^4^. The use of essential oils represents a proposed approach in food preservation that has been documented to effectively mitigate AMR^1^.

AMR bacterial infections are the cause of approximately 700,000 deaths worldwide, with projections indicating that this number will surge to 10 million by the year 2050^5,6^. For decades, the use of antibiotics has greatly reduced the mortality caused by various infectious diseases. However, their misuse or overuse has led to the emergence of antibiotic-resistant bacteria^7^. According to the WHO, *Salmonella* spp., *Enterobacteriaceae*, and *Staphylococcus aureus* are listed on the priority pathogen list for future research and development of new antibiotics^8^. This investment, with a specific emphasis on addressing gram-negative bacteria, represents an opportunity to combat AMR for an extended period, potentially spanning several decades^1,9^.

Studies had demonstrated the antimicrobial activity of numerous plant essential oils, including citrus limon^10^, lavender^11^, chicory^12^, and *Mentha pulegium* L.^13^. A plant with multifaceted effects is the date palm tree (*Phoenix dactylifera* L.), a member of the *Arecaceae* family encompassing over 1500 species across approximately 200 genera. Within this vast botanical family, the *Phoenix* palms represent a significant cluster, comprising 12 distinct species^14^. This plant has shown several biological activities including antioxidant, antimicrobial, anti-inflammatory, anti-hyperglycemic, anti-lipidemic, and anti-cancer activities^15^. Moreover, according to the Food and Agriculture Organization (FAO) in 2020, the worldwide production of date palm accounts for 8,526,218 metric tons (MT), with Asia and Africa being the leading regions (55.8% and 43.4%, respectively)^16^ with a substantial annual generation of date seed waste, which accounts for approximately 825,000 tons^16,17^. The seeds make up—10–15% of the date fruit weight depending on the cultivar^18^, and chemical composition differ due to differences in the type of cultivar, growing conditions, geographical region, stage of maturity, and the different analytical methods used for extraction and detection^19^. Despite the variation in chemical composition, date seeds have been shown to contain significantly high amounts of fiber, phenols and flavonoids, and relatively high antioxidant activities^20^. Date seeds have also been recently investigated for their valuable fat component, date seed oil^21^. The oil is considered to be oleic-lauric^22^ and has been shown to contain a remarkably high tocol content with dominancy to α-tocotrienol, followed by γ-tocopherol, γ-tocotrienol, δ-tocopherol, and β-tocopherol^23^. To the best of our knowledge, studies investigating the antimicrobial effects of date seed oil extracts are limited. The Medjoul date variety is the major cultivar grown in Jordan^24^. Accordingly, this study aimed to test the antimicrobial activity of Medjoul date seed oil against a number of selected pathogenic gram-negative and gram-positive bacteria in microbiological media at different storage temperatures (37 °C and 10 °C), and in the ultra-high temperature (UHT) milk as a food matrix at two different storage temperatures (10 °C and 4 °C).

## Results

### GC–MS analysis of date seed oil

To investigate whether temperature and time affect the antimicrobial activity of date seed oil, two sets of temperatures/times were chosen in this study. One set involved drying the date seeds at 50 °C for 5 h prior to oil extraction, and the other involved drying at 70 °C for 3 h. For simplification, the date seed oils (DSO) will be referred to as DSO (50 °C/5 h) and DSO (70 °C/3 h) respectively.

The antimicrobial effect of oils may result from a cascade of reactions rather than a unique mechanism and it is dependent on the chemical composition of the oil^25^. The GC–MS analysis for the two Medjoul DSO (50 °C/5 h and 70 °C/3 h) in the current study showed a total of 20 peaks for both oils including alkanes, fatty acids, aldehydes, fatty alcohols, and amides (Supplementary Figs. 1, 2). Supplementary Tables S1 and S2 list the retention times, compound names, and peak areas (%) of the detected compounds. The chemical constituents detected in DSO (50 °C/5 h) were 1.24% heptadecane, 1.29% 1-Nanodecene, 1.37% n-hexadecanoic acid, 2.39% tetradecane, 5.00% oleic acid, 6.60% pentadecane, 7.34% 9-Octadecenoic acid (Z)-, 8.54% heptane, and 55.43% octadecane. The chemical constituents detected in DSO (70 °C/3 h) were 1.35% 1-nanodecene, 1.66% glycidyl palmitate, 1.97% n-hexadecanoic acid, 2.13% tetradecane, 6.42% pentadecane, 10.95% 9-octadecenoic acid (Z)-, 13.36% oleic acid, and 52.22% octadecane.

### Fatty acid composition of date seed oil

Seventeen fatty acids were detected in both date seed oils (Supplementary Table S3). For the date seed oil (50 °C/5 h), the most abundant fatty acid was oleic acid (42.26%), followed by lauric acid (19.79%), myristic acid (10.67%), linoleic acid (9.82%), palmitic acid (9.23%), and stearic acid (3.52%). The order of fatty acids and their abundance were also similar for the date seed oil (70 °C/3 h) with slight differences in the percentages of oleic acid (43.06%), lauric acid (20.31%), myristic acid (10.67%), linoleic acid (9.78%), palmitic acid (9.11%), and stearic acid (3.68%).

### Antimicrobial activity of date seed oil

The antimicrobial activity of the two date seed oils against major foodborne pathogens was investigated at two different incubation temperatures, 37 °C for 24 h and 10 °C for 7 days (Table 1). At an incubation temperature of 37 °C for 24 h, DSO (50 °C/5 h) showed a significant bactericidal effect against the gram-negative bacteria *E. coli* O157:H7 (02:0627 and 02:0628), *S. enteritidis*, and *S. typhimurium* at a concentration of 15 µl/ml and reduced the counts by 6.7, 6.0, 4.3, and 6.0 logs, respectively. The numbers of gram-positive bacteria *St. aureus* ATCC 33591 and ATCC 43300 under the same incubation conditions were reduced by 2.0 and 5.0 log, respectively, at the same concentration. However, DSO (70°C/3h) showed a slightly higher inhibition against all strains compared to DSO (50°C/5h), where at a concentration of 15 µl/ml, the oil (treatment at 70 °C for 3 h) reduced the numbers of *E. coli* O157:H7 (02:0627), *St. aureus* (ATCC 33591), *St. aureus* (ATCC 43300), *S. enteritidis*, and *S. typhimurium* by 6.0, 3.5, 6.0, 4.5, and 7.0 logs respectively, and the numbers of *E. coli* O157: H7 (02:0628) were completely eliminated at concentrations of 10µl/ml and 15µl/ml. On the other hand, both oil treatments showed a lower inhibition concentration after incubation at 10 °C for 7 days compared to the previous incubation conditions. For instance, both treatments resulted in a reduction of *E. coli* O157:H7 (02:0627) count by 3.8–5.1 logs at different concentrations of 5–15 µl/ml. Similarly, the reduction of *St. aureus* ATCC 33591 by DSO (50°C/5h) and DSO (70 °C/3 h) reached 1.5 logs and 2 log, respectively, at a concentration of 5–10 µl/ml at 10 °C. While for *St. aureus* ATCC 43300, DSO (50 °C/5 h) and DSO (70 °C/3 h) resulted in 1.0 log and 1.4 log reductions at a concentration of 5 µl/ml and 2.5 µl/ml, respectively. However, in both *St. aureus* strains, higher concentrations led to a decrease in the log reduction. Contrary to previous data, the results indicated that both date seed oils had little to no antimicrobial effect against *L. monocytogenes* strains. Among the two *L. monocytogenes* strains, the highest log reduction was observed for DSO (50 °C/5 h) against *L. monocytogenes* ATCC 19115 at an incubation temperature of 37 °C, which was approximately 0.64 logs.

**Table 1:** Log numbers for the different foodborne pathogens after treatment with date seed oil and control.

Bacterial strain	Control and concentration of DSO	37 °C for 24 h		10 °C for 7 days	
		DSO (50 °C)	DSO (70 °C)	DSO (50 °C)	DSO (70 °C)
*Escherichia coli* O157:H7 (02:0627)	Control	${9.06 \pm 0.01}^{\mathrm{f}}$		${8.37 \pm 0.13}^{\mathrm{j}}$	
	15 µl/ml	${2.33 \pm 0.15}^{\mathrm{a}}$	${3.10 \pm 0.10}^{\mathrm{b}}$	${4.46 \pm 0.02}^{\mathrm{ef}}$	${3.57 \pm 0.03}^{\mathrm{ab}}$
	10 µl/ml	${7.59 \pm 0.06}^{\mathrm{cd}}$	${7.10 \pm 0.10}^{\mathrm{c}}$	${4.56 \pm 0.01}^{\mathrm{ef}}$	${3.72 \pm 0.02}^{\mathrm{bc}}$
	5 µl/ml	${7.69 \pm 0.39}^{\mathrm{cd}}$	${7.77 \pm 0.04}^{\mathrm{cd}}$	${3.26 \pm 0.14}^{\mathrm{a}}$	${4.01 \pm 0.06}^{\mathrm{cd}}$
	2.5 µl/ml	${8.22 \pm 0.01}^{\mathrm{de}}$	${8.27 \pm 0.10}^{\mathrm{de}}$	${5.06 \pm 0.07}^{\mathrm{g}}$	${4.28 \pm 0.06}^{\mathrm{de}}$
	1.25 µl/ml	${8.64 \pm 0.06}^{\mathrm{ef}}$	${8.54 \pm 0.06}^{\mathrm{ef}}$	${6.31 \pm 0.10}^{\mathrm{h}}$	${4.28 \pm 0.09}^{\mathrm{de}}$
	0.625 µl/ml	${8.88 \pm 0.07}^{\mathrm{ef}}$	${8.83 \pm 0.05}^{\mathrm{ef}}$	${7.87 \pm 0.03}^{\mathrm{i}}$	${4.84 \pm 0.04}^{\mathrm{fg}}$
*Escherichia coli* O157:H7 (02:0628)	Control	${9.23 \pm 0.01}^{\mathrm{d}}$		${6.55 \pm 0.06}^{\mathrm{f}}$	
	15 µl/ml	${3.31 \pm 0.13}^{\mathrm{b}}$	${0.00 \pm 0.00}^{\mathrm{a}}$	${3.43 \pm 0.10}^{\mathrm{b}}$	${2.74 \pm 0.02}^{\mathrm{a}}$
	10 µl/ml	${3.56 \pm 0.04}^{\mathrm{b}}$	${0.00 \pm 0.00}^{\mathrm{a}}$	${3.62 \pm 0.26}^{\mathrm{b}}$	${2.48 \pm 0.00}^{\mathrm{a}}$
	5 µl/ml	${7.83 \pm 0.02}^{\mathrm{c}}$	${3.85 \pm 0.04}^{\mathrm{b}}$	${4.19 \pm 0.09}^{\mathrm{c}}$	${2.54 \pm 0.04}^{\mathrm{a}}$
	2.5 µl/ml	${8.28 \pm 0.64}^{\mathrm{cd}}$	${3.78 \pm 0.15}^{\mathrm{b}}$	${4.60 \pm 0.03}^{\mathrm{c}}$	${3.35 \pm 0.18}^{\mathrm{b}}$
	1.25 µl/ml	${8.50 \pm 0.11}^{\mathrm{cd}}$	${8.81 \pm 0.02}^{\mathrm{cd}}$	${5.29 \pm 0.05}^{\mathrm{d}}$	${4.31 \pm 0.01}^{\mathrm{c}}$
	0.625 µl/ml	${8.73 \pm 0.01}^{\mathrm{cd}}$	${8.82 \pm 0.05}^{\mathrm{d}}$	${6.03 \pm 0.06}^{\mathrm{e}}$	${5.47 \pm 0.01}^{\mathrm{d}}$
*Salmonella entritidis*	Control	${9.34 \pm 0.05}^{\mathrm{g}}$		${7.61 \pm 0.06}^{\mathrm{d}}$	
	15 µl/ml	${5.05 \pm 0.01}^{\mathrm{a}}$	${4.90 \pm 0.01}^{\mathrm{a}}$	${3.30 \pm 0.17}^{\mathrm{a}}$	${3.74 \pm 0.22}^{\mathrm{a}}$
	10 µl/ml	${6.54 \pm 0.03}^{\mathrm{bc}}$	${6.69 \pm 0.04}^{\mathrm{cd}}$	${5.67 \pm 0.03}^{\mathrm{b}}$	${5.53 \pm 0.12}^{\mathrm{b}}$
	5 µl/ml	${6.23 \pm 0.17}^{\mathrm{b}}$	${6.72 \pm 0.12}^{\mathrm{cd}}$	${6.59 \pm 0.10}^{\mathrm{bcd}}$	${5.67 \pm 0.67 }^{\mathrm{b}}$
	2.5 µl/ml	${6.90 \pm 0.04}^{\mathrm{cd}}$	${7.02 \pm 0.12}^{\mathrm{d}}$	${6.23 \pm 0.01}^{\mathrm{bc}}$	${6.24 \pm 0.06}^{\mathrm{bc}}$
	1.25 µl/ml	${8.06 \pm 0.02}^{\mathrm{ef}}$	${7.93 \pm 0.03}^{\mathrm{e}}$	${6.92 \pm 0.01}^{\mathrm{cd}}$	${6.99 \pm 0.02}^{\mathrm{cd}}$
	0.625 µl/ml	${8.36 \pm 0.08}^{\mathrm{f}}$	${8.12 \pm 0.03}^{\mathrm{ef}}$	${7.48 \pm 0.05}^{\mathrm{d}}$	${7.38 \pm 0.00}^{\mathrm{d}}$
*Salmonella typhimurium*	Control	${9.34 \pm 0.13}^{\mathrm{e}}$		${7.85 \pm 0.00}^{\mathrm{g}}$	
	15 µl/ml	${3.15 \pm 1.01}^{\mathrm{a}}$	${2.43 \pm 0.30}^{\mathrm{a}}$	${3.67 \pm 0.05}^{\mathrm{ab}}$	${4.08 \pm 0.05}^{\mathrm{abc}}$
	10 µl/ml	${3.67 \pm 0.76}^{\mathrm{a}}$	${3.10 \pm 0.38}^{\mathrm{a}}$	${3.15 \pm 0.09}^{\mathrm{a}}$	${5.00 \pm 0.00}^{\mathrm{cd}}$
	5 µl/ml	${6.90 \pm 0.02}^{\mathrm{cd}}$	${4.09 \pm 0.09}^{\mathrm{ab}}$	${4.49 \pm 0.51}^{\mathrm{bc}}$	${4.19 \pm 0.44}^{\mathrm{bc}}$
	2.5 µl/ml	${8.59 \pm 0.04}^{\mathrm{de}}$	${4.39 \pm 0.06}^{\mathrm{ab}}$	${6.05 \pm 0.03}^{\mathrm{e}}$	${5.82 \pm 0.02}^{\mathrm{de}}$
	1.25 µl/ml	${8.73 \pm 0.07}^{\mathrm{de}}$	${6.03 \pm 0.51}^{\mathrm{bc}}$	${6.79 \pm 0.00}^{\mathrm{ef}}$	${6.50 \pm 0.06}^{\mathrm{ef}}$
	0.625 µl/ml	${9.17 \pm 0.05 }^{\mathrm{e}}$	8.95 ± 0.02^de^	${7.28 \pm 0.03}^{\mathrm{fg}}$	${6.79 \pm 0.03}^{\mathrm{ef}}$
*Staphylococcus aureus *ATCC 33591	Control	${9.29 \pm 0.06}^{\mathrm{fg}}$		${5.26 \pm 0.28}^{\mathrm{e}}$	
	15 µl/ml	${7.10 \pm 0.01}^{\mathrm{b}}$	${5.79 \pm 0.06}^{\mathrm{a}}$	${4.71 \pm 0.04}^{\mathrm{ef}}$	${4.65 \pm 0.01}^{\mathrm{cde}}$
	10 µl/ml	${7.42 \pm 0.06}^{\mathrm{b}}$	${7.30 \pm 0.30}^{\mathrm{b}}$	${3.73 \pm 0.03}^{\mathrm{ab}}$	${3.47 \pm 0.23}^{\mathrm{a}}$
	5 µl/ml	${8.21 \pm 0.02}^{\mathrm{c}}$	${8.37 \pm 0.05}^{\mathrm{cd}}$	${3.95 \pm 0.21}^{\mathrm{abc}}$	${3.36 \pm 0.18}^{\mathrm{a}}$
	2.5 µl/ml	${8.60 \pm 0.03}^{\mathrm{cde}}$	${8.54 \pm 0.07}^{\mathrm{cd}}$	${4.24 \pm 0.04}^{\mathrm{bcd}}$	${3.36 \pm 0.18}^{\mathrm{a}}$
	1.25 µl/ml	${9.08 \pm 0.02}^{\mathrm{efg}}$	${8.76 \pm 0.01}^{\mathrm{de}}$	${4.53 \pm 0.08}^{\mathrm{cde}}$	${3.59 \pm 0.06}^{\mathrm{ab}}$
	0.625 µl/ml	${9.39 \pm 0.06}^{\mathrm{g}}$	${8.84 \pm 0.01}^{\mathrm{def}}$	${4.82 \pm 0.06}^{\mathrm{cde}}$	${3.75 \pm 0.03}^{\mathrm{ab}}$
*Staphylococcus aureus *ATCC 43300	Control	${9.15 \pm 0.02}^{\mathrm{h}}$		${5.44 \pm 0.13}^{\mathrm{i}}$	
	15 µl/ml	${4.04 \pm 0.05}^{\mathrm{b}}$	${3.06 \pm 0.06}^{\mathrm{a}}$	${4.82 \pm 0.10}^{\mathrm{h}}$	${4.72 \pm 0.03}^{\mathrm{f}}$
	10 µl/ml	${5.59 \pm 0.15}^{\mathrm{c}}$	${5.36 \pm 0.18}^{\mathrm{c}}$	${4.69 \pm 0.05}^{\mathrm{fgh}}$	${4.36 \pm 0.02}^{\mathrm{bcde}}$
	5 µl/ml	${6.90 \pm 0.01}^{\mathrm{d}}$	${5.68 \pm 0.08}^{\mathrm{c}}$	${4.30 \pm 0.04}^{\mathrm{abcd}}$	${4.12 \pm 0.01}^{\mathrm{ab}}$
	2.5 µl/ml	${7.52 \pm 0.04}^{\mathrm{e}}$	${8.39 \pm 0.10}^{\mathrm{f}}$	${4.39 \pm 0.01}^{\mathrm{bcde}}$	${4.05 \pm 0.03}^{\mathrm{a}}$
	1.25 µl/ml	${8.33 \pm 0.02}^{\mathrm{f}}$	${8.63 \pm 0.01}^{\mathrm{fg}}$	${4.53 \pm 0.01}^{\mathrm{defg}}$	${4.23 \pm 0.06}^{\mathrm{abc}}$
	0.625 µl/ml	${8.96 \pm 0.03}^{\mathrm{gh}}$	${8.52 \pm 0.04}^{\mathrm{f}}$	${4.58 \pm 0.03}^{\mathrm{efgh}}$	${4.43 \pm 0.04}^{\mathrm{cdef}}$
*Listeria monocytogenes* ATCC 7644	Control	${8.74 \pm 0.03}^{\mathrm{f}}$		${8.97 \pm 0.07}^{\mathrm{abc}}$	
	15 µl/ml	${8.33 \pm 0.04}^{\mathrm{a}}$	${8.45 \pm 0.02}^{\mathrm{ab}}$	${9.08 \pm 0.02}^{\mathrm{bcd}}$	${8.84 \pm 0.02}^{\mathrm{a}}$
	10 µl/ml	${8.44 \pm 0.02}^{\mathrm{ab}}$	${8.51 \pm 0.01}^{\mathrm{bcd}}$	${9.00 \pm 0.06}^{\mathrm{abcd}}$	${8.89 \pm 0.04}^{\mathrm{ab}}$
	5 µl/ml	${8.48 \pm 0.05}^{\mathrm{bc}}$	${8.53 \pm 0.00}^{\mathrm{bcde}}$	${9.05 \pm 0.01}^{\mathrm{bcd}}$	${9.07 \pm 0.06}^{\mathrm{bcd}}$
	2.5 µl/ml	${8.53 \pm 0.02}^{\mathrm{bcde}}$	${8.57 \pm 0.01}^{\mathrm{bcde}}$	${9.04 \pm 0.02}^{\mathrm{abcd}}$	${9.06 \pm 0.03}^{\mathrm{bcd}}$
	1.25 µl/ml	${8.55 \pm 0.05}^{\mathrm{bcde}}$	${8.61 \pm 0.01}^{\mathrm{cdef}}$	${9.05 \pm 0.01}^{\mathrm{bcd}}$	${9.12 \pm 0.06}^{\mathrm{cd}}$
	0.625 µl/ml	${8.64 \pm 0.01}^{\mathrm{def}}$	${8.66 \pm 0.02}^{\mathrm{ef}}$	${9.05 \pm 0.01}^{\mathrm{abcd}}$	${9.18 \pm 0.00}^{\mathrm{d}}$
*Listeria monocytogenes* ATCC 19115	Control	${9.19 \pm 0.09}^{\mathrm{g}}$		${9.12 \pm 0.10}^{\mathrm{bc}}$	
	15 µl/ml	${8.99 \pm 0.01}^{\mathrm{ef}}$	${9.08 \pm 0.05}^{\mathrm{fg}}$	${8.85 \pm 0.01}^{\mathrm{ab}}$	${8.77 \pm 0.02}^{\mathrm{a}}$
	10 µl/ml	${8.55 \pm 0.06}^{\mathrm{a}}$	${8.92 \pm 0.00}^{\mathrm{cdef}}$	${8.95 \pm 0.03}^{\mathrm{abc}}$	${8.90 \pm 0.04}^{\mathrm{abc}}$
	5 µl/ml	${8.67 \pm 0.03}^{\mathrm{ab}}$	${8.75 \pm 0.02}^{\mathrm{bc}}$	${8.99 \pm 0.10}^{\mathrm{abc}}$	${8.94 \pm 0.05}^{\mathrm{abc}}$
	2.5 µl/ml	${8.77 \pm 0.01}^{\mathrm{bcd}}$	${8.89 \pm 0.03}^{\mathrm{cde}}$	${8.99 \pm 0.04}^{\mathrm{abc}}$	${8.95 \pm 0.03}^{\mathrm{abc}}$
	1.25 µl/ml	${8.79 \pm 0.03}^{\mathrm{bcd}}$	${8.92 \pm 0.01}^{\mathrm{cdef}}$	${8.96 \pm 0.01}^{\mathrm{abc}}$	${8.95 \pm 0.06}^{\mathrm{abc}}$
	0.625 µl/ml	${8.86 \pm 0.01}^{\mathrm{bcde}}$	${8.87 \pm 0.03}^{\mathrm{def}}$	${9.04 \pm 0.02}^{\mathrm{bc}}$	${9.08 \pm 0.03}^{\mathrm{bc}}$

Data are shown as the mean ± SEM (n = 3).

*DSO* date seed oil.

For each group, the means of the treatments were compared to their respective controls used for the same bacterial strains and the same conditions, with different letters indicating significant differences (p < 0.05).

### Antimicrobial activity of date seed oil in UHT skim milk

**Figure 1:**
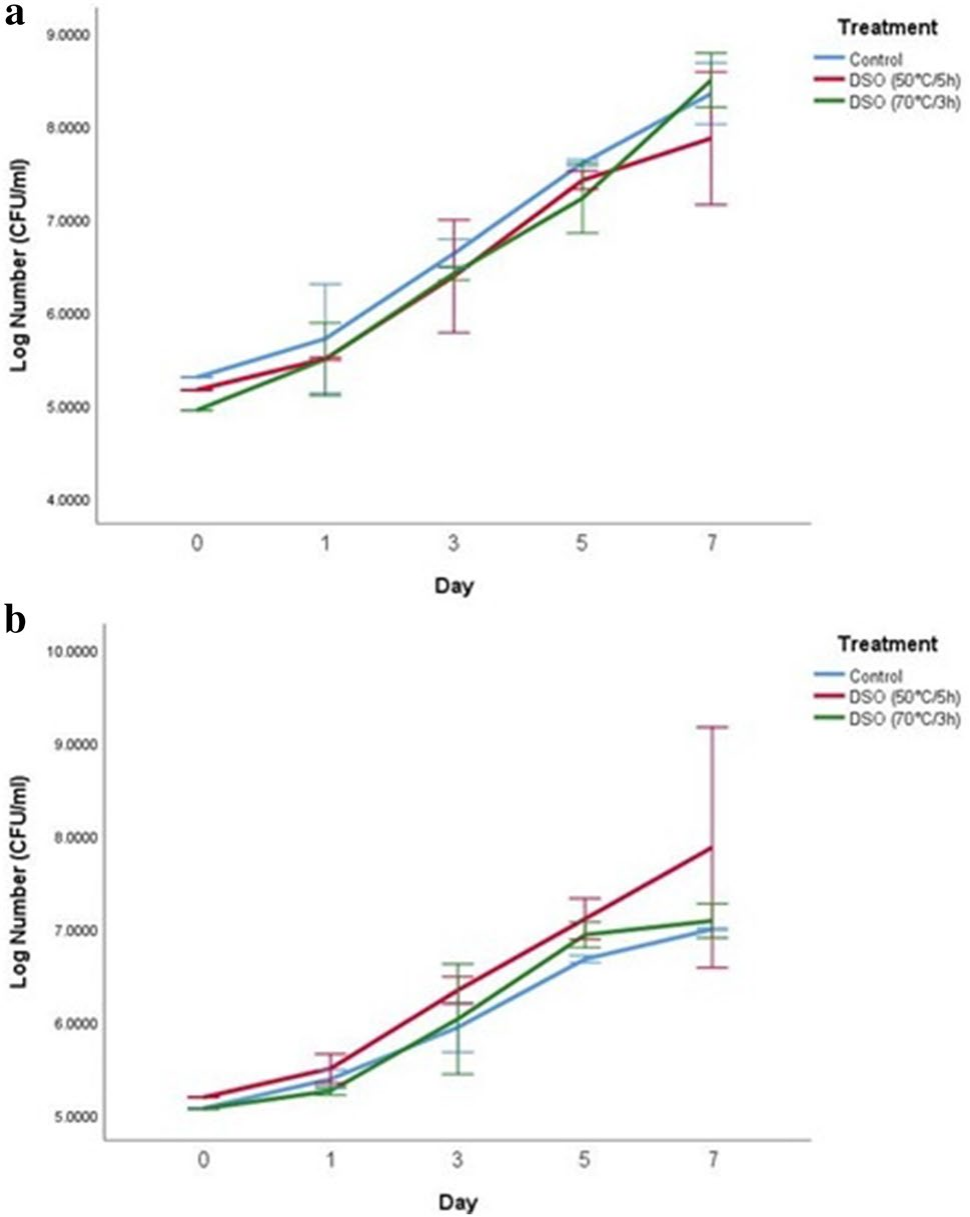
Log number of *St. aureus* on days 0, 1, 3, 5, and 7 with control and two treatments of date seed oil (50 °C/5h) and (70 °C/3 h) in UHT Skim milk. The concentration used for both oils was 15 µl/ml. (**a**) Incubation at 10 °C; (**b**) incubation at 4 °C; *DSO* date seed oil.

**Figure 2:**
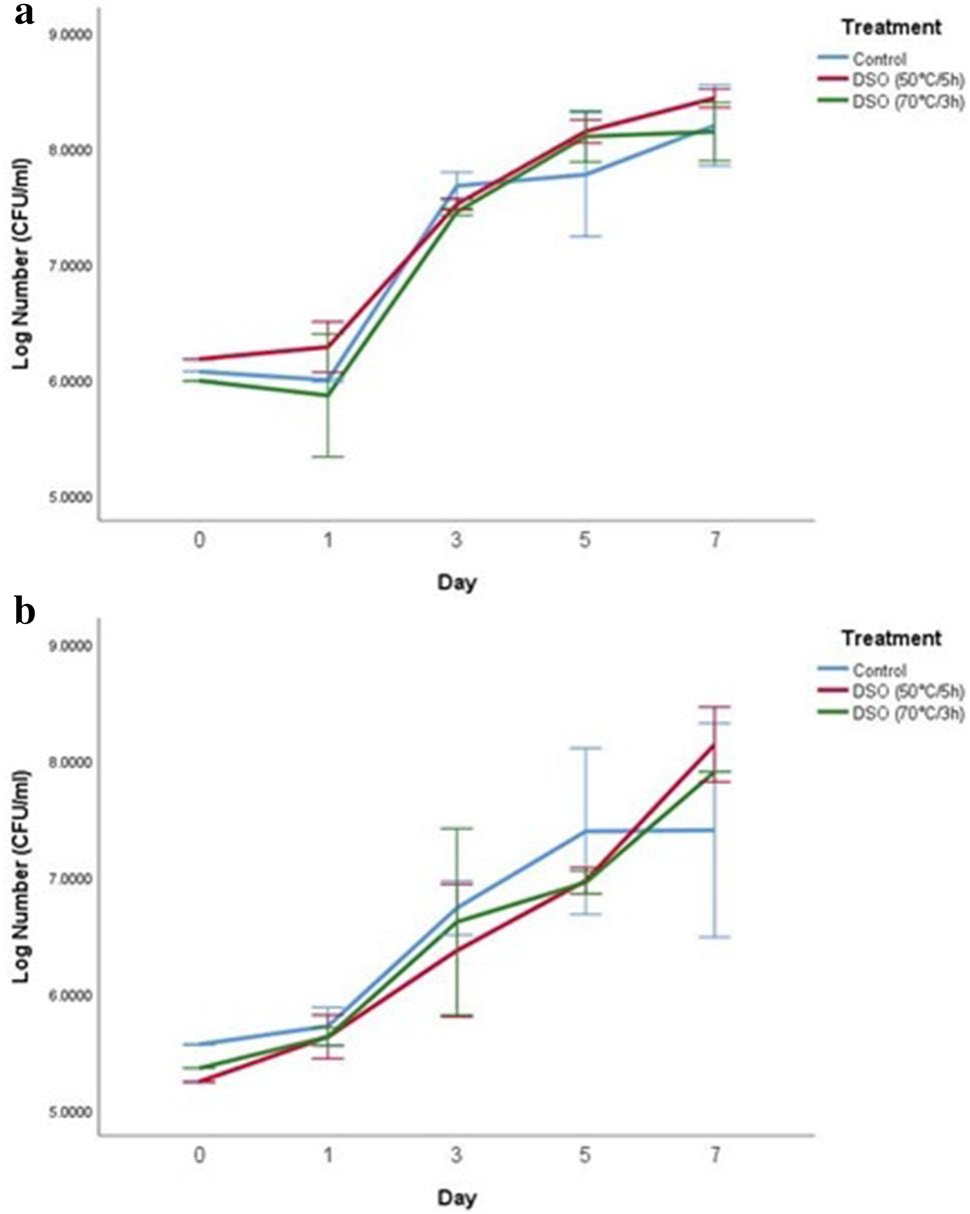
Log number of *L. monocytogenes* on days 0, 1, 3, 5, and 7 with control and two treatments of date seed oil (50 °C/5h) and (70 °C/3 h) in UHT Skim milk. The concentration used for both oils was 15 µl/ml. (**a**) Incubation at 10 °C; (**b**) incubation at 4 °C. *DSO* date seed oil.

**Figure 3:**
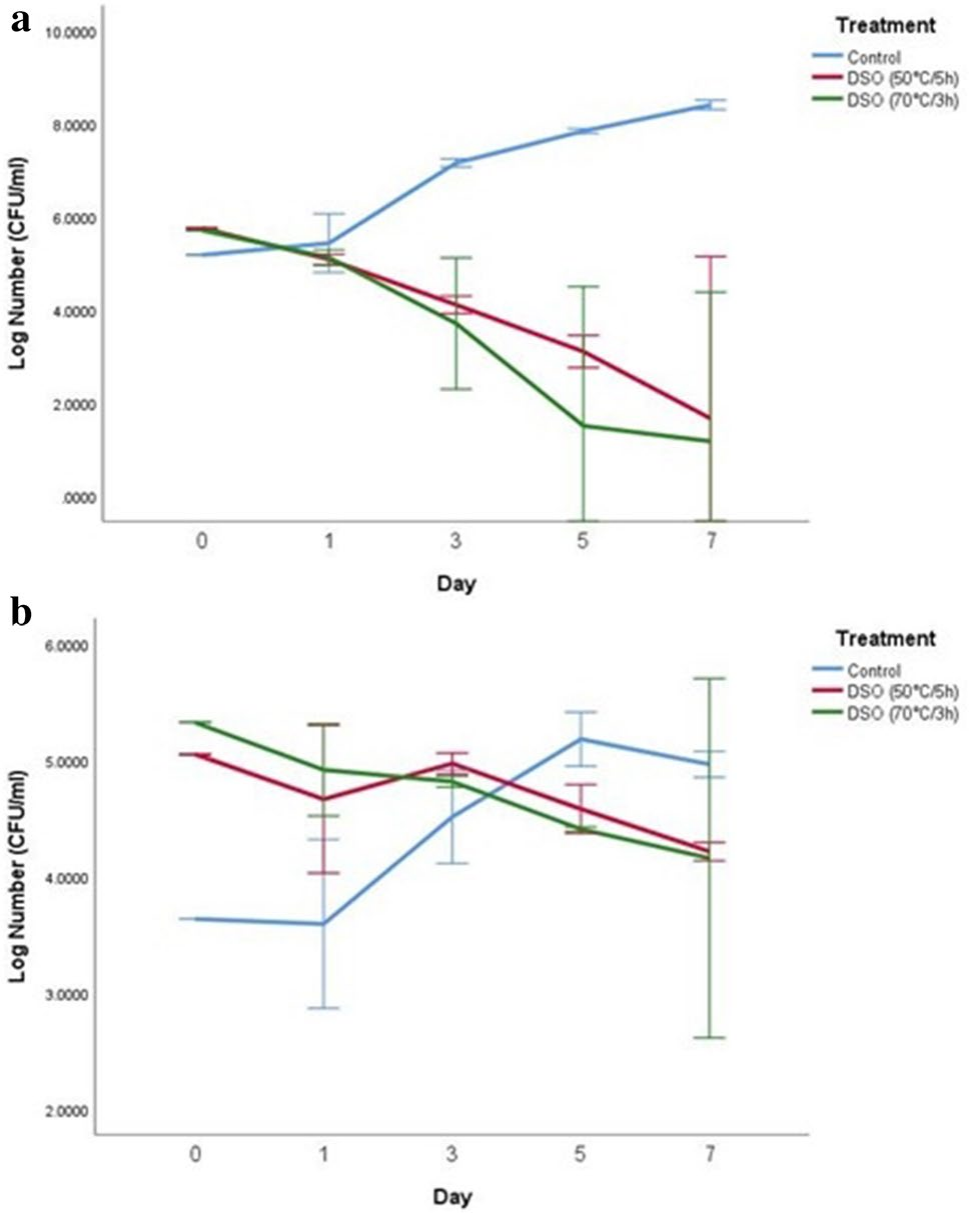
Log number of *Salmonella* spp. on days 0, 1, 3, 5, and 7 with control and two treatments of date seed oil (50 °C/5 h) and (70 °C/3 h) in UHT Skim milk. The concentration used for both oils was 15 µl/ml. (**a**) Incubation at 10 °C; (**b**) incubation at 4 °C; *DSO* date seed oil.

**Figure 4:**
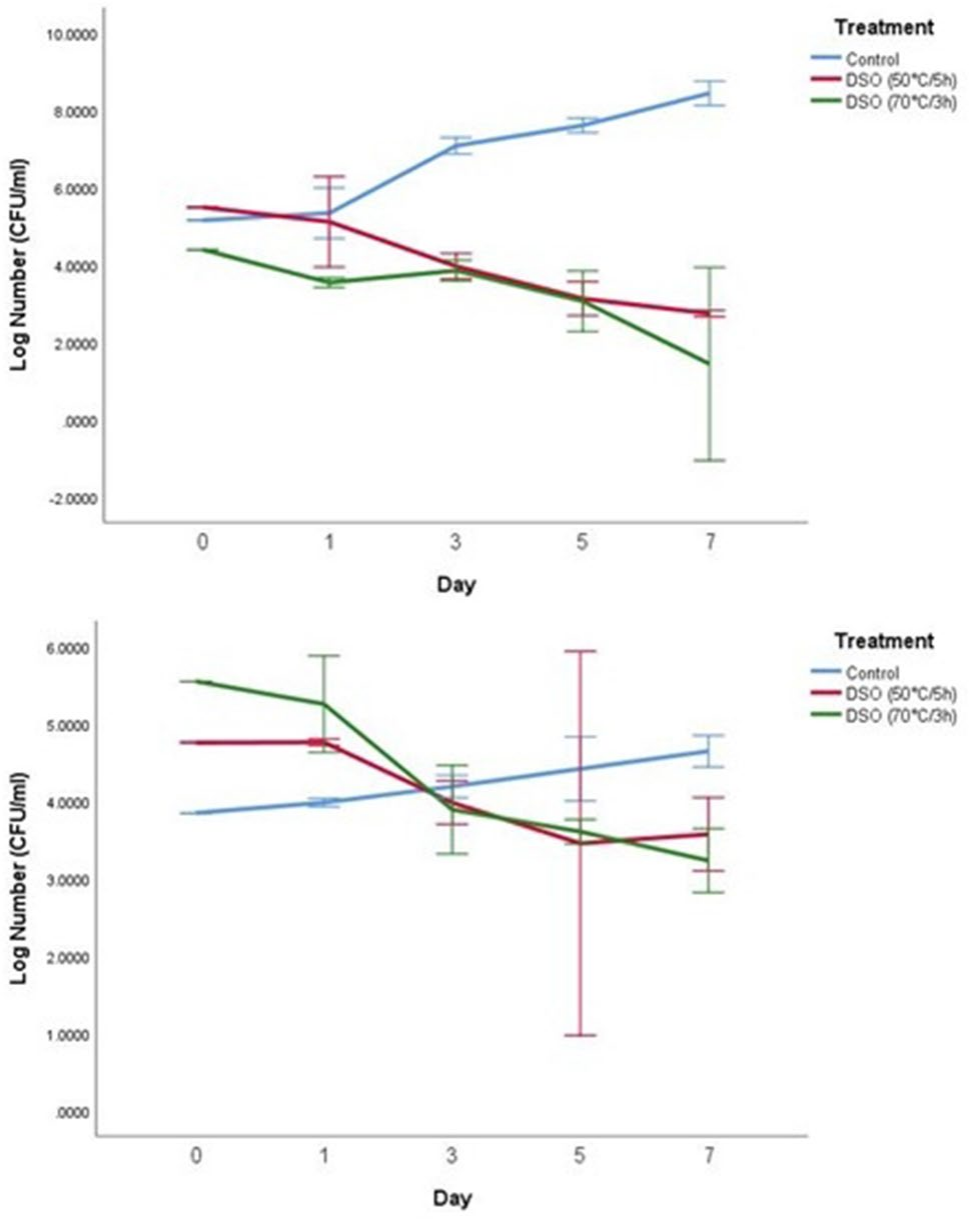
Log number of *E. coli* O157:H7 on days 0, 1, 3, 5, and 7 with control and two treatments of date seed oil (50 °C/5 h) and (70 °C/3 h) in UHT skim milk. The concentration used for both oils was 15 µl/ml. (**a**) Incubation at 10 °C; (**b**) incubation at 4 °C; *DSO* date seed oil.

Given that food matrices are composed of several different food ingredients, various molecular interactions can occur when an antimicrobial is added, which could affect its behavior in the food matrix when compared with microbiological media^26,27^. In this study, UHT-skim milk stored at 10 °C and 4 °C for 7 days was used as a food model system (in situ) to evaluate the efficiency of date seed oil at a concentration of 15 µl/ml. The results showed that the addition of date seed oil to UHT-skim milk did not inhibit the growth of the gram-positive bacteria *St. aureus* and *L. monocytogenes* at 10 °C and 4 °C (Figs. 1, 2). In contrast, date seed oil had a significant inhibitory effect against gram-negative bacteria including *Salmonella* and *E. coli* O157:H7 at 10 °C and a slight inhibition at 4 °C. For DSO (50 °C/5 h), log reductions of approximately 6.8 and 5.7 for *Salmonella* and *E. coli* O157:H7, respectively, were observed by the end of the storage period at an incubation temperature of 10 °C. However, at a storage temperature of 4 °C, DSO (50 °C/5 h) resulted in a lower log reduction of approximately 0.8 logs and 1.1 logs for *Salmonella* and *E. coli* O157:H7, respectively. However, a higher inhibitory effect was observed for DSO (70°C/3h) at a storage temperature of 10 °C, resulting in 7.2 logs and 7.0 logs reduction in *Salmonella* and *E. coli* O157: H7, respectively, after 7 days. Similarly, at a storage temperature of 4 °C, the DSO (70 °C/3 h) had a weaker inhibitory effect than the higher storage temperature (10 °C) and resulted in log reductions of approximately 0.8 logs and 2 logs for *Salmonella* and *E. coli* O157:H7, respectively (Figs. 3, 4).

### Morphological changes in bacterial cells

**Figure 5:**
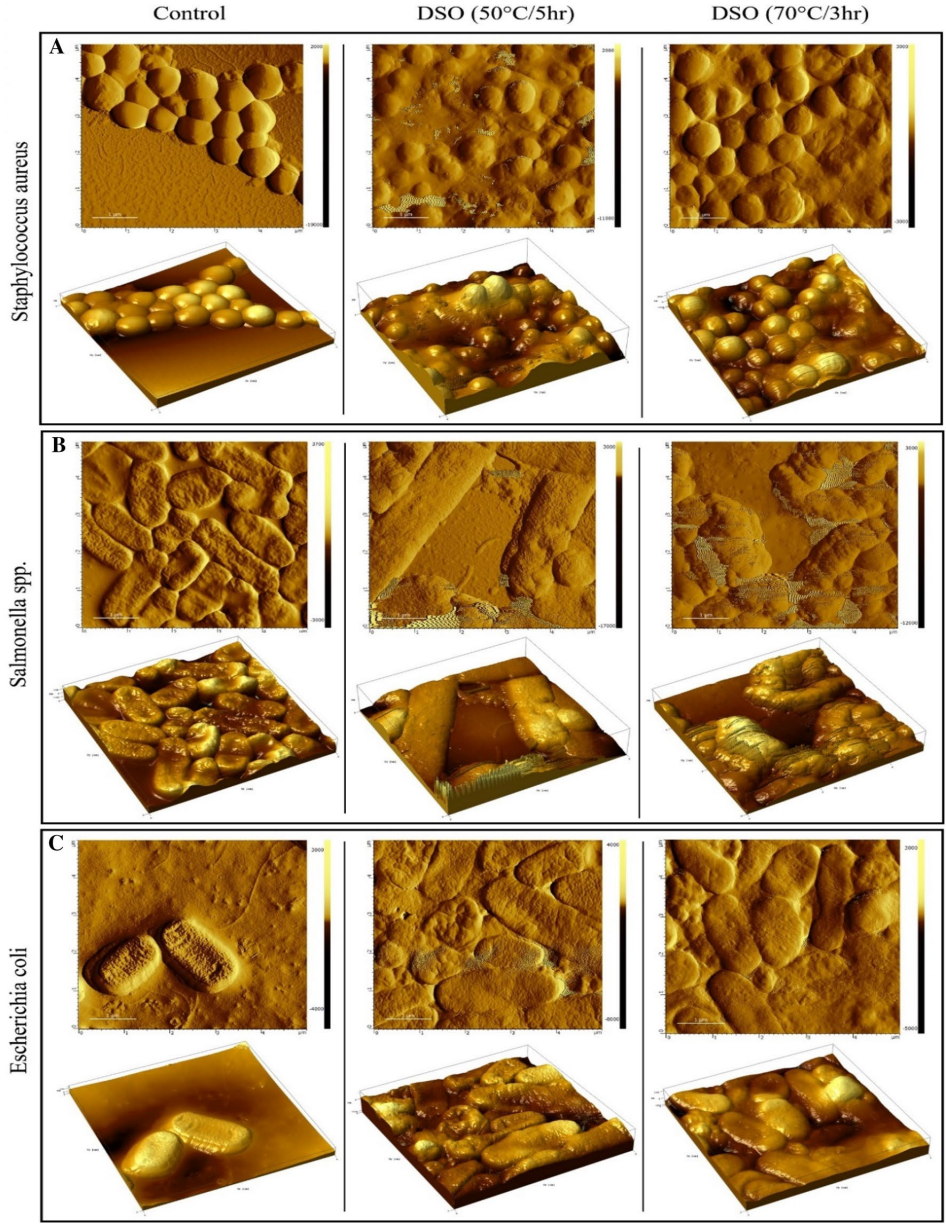
2D and 3D atomic force microscopy (AFM) images of (**A**) *Staphylococcus aureus*, (**B**) *Salmonella* spp., and (**C**) *Escherichia coli* control and date seed oil treated cells. Scan area 5 µm × 5 µm.

Imaging of bacteria was performed using atomic force microscopy (AFM), which is a powerful tool that allows the study of microbial surfaces and changes in structure at nanometer scale^28^. The non-contact mode was applied in this study, with the cantilever maintaining a distance of tens to hundreds of angstroms (Å) away from the sample and only vibrating near it^29^, which protects the sample and the tip from damage and is considered one of the main advantages of using the non-contact mode instead of the contact mode^30^. The purpose of using AFM in our study was to examine morphological changes in the cells and to gain insight into the mechanism of action of DSO. As shown in Fig. 5, control images revealed bacterial surfaces that were smooth, where *Staphylococcus* cocci cells were arranged in clusters; a typical morphology characteristic of *St. aureus*, and gram-negative bacteria (i.e., *E. coli* and *Salmonella* spp.) maintained a characteristic rod-like shape. In contrast, when treated with DSO (10µl/ml), *St. aureus* cells showed a loss of cluster arrangement, and the bacteria had distorted shapes, surface blebs (protrusions), indentations, stiffness, and swelling (particularly in gram-negative cells). To analyze the morphological changes in more detail, AFM images were analyzed using the Gwyddion Software (Table 2). According to these results, *St. aureus* cells treated with DSO showed a decrease in length and width compared with the control. In contrast, *E. coli* and *Salmonella* spp. cells showed an increase in length and width. The average roughness and root mean square roughness were shown to decrease in all treated samples, except for *Salmonella* spp. treated with DSO (70°C/3 h).

**Table 2:** Bacterial cell dimensions and cell roughness for the different foodborne pathogens after treatment with date seed oil and control.

Bacteria	Length (µm)	Width (µm)	Height (µm)	Roughness average (nm)	Root mean square roughness (nm)
*St. aureus*, control	0.78 ± ${0.06}^{\mathrm{a}}$	0.78 ± ${0.07}^{\mathrm{a}}$	0.34 ± ${0.08}^{\mathrm{a}}$	21.40 ± ${7.56}^{\mathrm{a}}$	26.35 ± ${11.00}^{\mathrm{a}}$
*St. aureus* + DSO (50 °C/5h)	0.63 ± ${0.10}^{\mathrm{a}}$	0.58 ± ${0.05}^{\mathrm{b}}$	0.13 ± ${0.03}^{\mathrm{b}}$	10.46 ± ${3.41}^{\mathrm{b}}$	12.47 ± ${4.29}^{\mathrm{b}}$
*St. aureus* + DSO (70 °C/3h)	0.67 ± ${0.10}^{\mathrm{a}}$	0.71 ± ${0.10}^{\mathrm{ab}}$	0.11 ± ${0.02}^{\mathrm{b}}$	6.47 ± ${1.53}^{\mathrm{b}}$	7.97 ± ${1.84}^{\mathrm{b}}$
*Salmonella* spp., control	1.83 ± ${0.23}^{\mathrm{a}}$	0.78 ± ${0.08}^{\mathrm{a}}$	0.15 ± ${0.05}^{\mathrm{a}}$	5.20 ± ${1.24}^{\mathrm{a}}$	6.92 ± ${1.74}^{\mathrm{a}}$
*Salmonella* spp. + DSO (50 °C/5h)	3.05 ± ${1.16}^{\mathrm{a}}$	1.08 ± ${0.09}^{\mathrm{b}}$	0.23 ± ${0.10}^{\mathrm{a}}$	2.08 ± ${0.22}^{\mathrm{a}}$	3.00 ± ${0.50}^{\mathrm{b}}$
*Salmonella* spp. + DSO (70 °C/3h)	2.82 ± ${0.46}^{\mathrm{a}}$	0.96 ± ${0.16}^{\mathrm{ab}}$	0.27 ± ${0.06}^{\mathrm{a}}$	13.01 ± ${2.57}^{\mathrm{b}}$	16.87 ± ${3.18}^{\mathrm{b}}$
*E. coli* O157:H7, control	1.91 ± ${0.26}^{\mathrm{a}}$	1.21 ± ${0.01}^{\mathrm{a}}$	0.13 ± ${0.02}^{\mathrm{a}}$	3.99 ± ${0.62}^{\mathrm{a}}$	5.51 ± ${0.59}^{\mathrm{a}}$
*E. coli* O157:H7 + DSO (50 °C/5h)	2.23 ± ${0.67}^{\mathrm{a}}$	1.01 ± ${0.22}^{\mathrm{a}}$	0.11 ± ${0.03}^{\mathrm{a}}$	3.85 ± ${1.54}^{\mathrm{a}}$	5.46 ± ${2.48}^{\mathrm{a}}$
*E. coli* O157:H7 + DSO (70 °C/3h)	1.96 ± ${0.48}^{\mathrm{a}}$	0.91 ± ${0.13}^{\mathrm{a}}$	0.13 ± ${0.02}^{\mathrm{a}}$	2.80 ± ${0.75}^{\mathrm{a}}$	3.81 ± ${1.08}^{\mathrm{a}}$

Data are shown as the mean ± SEM (n = 3).

*DSO* date seed oil.

For each group, the means of the treatments were compared to their respective controls used for the same bacterial strains and the same conditions, with different letters indicating significant differences (p < 0.05).

## Discussion

Traditionally, synthetic preservatives have played a crucial role in the food industry by effectively preventing microbial growth and lipid oxidation^31^. However, the use of these preservatives has raised significant concerns owing to their associated health risks, including allergies, hyperactivity, cancer, and neurological damage^32^. Moreover, these preservatives have been reported to be a potential stress factor that could contribute to AMR^3,33^. Consequently, there has been a notable shift in the demand for safer and more natural food products by both food manufacturers and consumers, driven by increasing awareness of the potential health hazards posed by synthetic preservatives and the demand for minimally processed foods, especially those containing natural preservatives^2,34^. The use of phytochemicals such as essential oils has demonstrated efficacy in various applications by reducing the growth and viability of microorganisms, making them suitable alternatives to antibiotics^35^. For instance, in a study carried out by Alizadeh Behbahani et al.^12^, chicory essential oil (CEO) was incorporated into *Lepidium perfoliatum* seed mucilage to develop an edible coating with rich antioxidant and antimicrobial properties Alizadeh Behbahani et al.^12^. The successful application of this edible coating was evident through the notable reduction in microbial growth, lipid oxidation, texture alteration, and weight loss in the tested beef slices compared to their oil-free counterparts. This outcome ultimately enhanced the shelf life of the food product through the use of natural compounds^12^. Likewise, essential oils like citrus limon and Mentha pulegium, when combined with different seed mucilages, have exhibited enhanced antimicrobial properties, and prolonged the shelf life of perishable meat products^10,13^. Thus, these findings demonstrate the effective utilization of natural phytochemicals as antimicrobial agents for the preservation of food products.

Previous studies have investigated the antimicrobial activity of aqueous, ethanolic, and methanolic extracts derived from date seeds. However, the antimicrobial potential of date seed oil has been relatively underexplored. A study investigating the antimicrobial effect of Nigerian date seed cultivars against strains of *S. aureus*, Majekodunmi et al.^36^ used two types of extracts (aqueous and ethanolic) and reported the same MIC values of 20 mg/ml and with 80 mg/ml MBC values for both extracts. Despite having the same values, Majekodunmi et al.^36^ observed that the ethanolic extract exhibited superior efficacy based on the inhibition zones previously determined. Specifically, the ethanolic extract demonstrated an inhibition zone of 12 mm at a concentration of 100 mg/ml, whereas the aqueous extract only achieved an inhibition zone of 8mm. This discrepancy was attributed to the active ingredients present in the date seeds which could have been more soluble in ethanol than in water. Similar findings were obtained by Chinelo et al.^37^, when investigating the effect of a methanolic seed extract against several *E. coli* and reported a MIC value ranging between 10 and 19.9 mg/ml. On the other hand, Shakiba et al.^38^ conducted a study using a methanolic extract to evaluate the antimicrobial effect of an Iranian cultivar against three *St. aureus* strains and one *E.coli* strain. According to the results, one *E. coli* strain along with two of the *S. aureus* strains exhibited identical MIC values of 1.25 mg/ml and an MBC values of 2.5 mg/ml. In contrast, the third strain displayed an MIC value of 2.5 mg/ml and an MBC value of 5 mg/ml. These findings underscore the potential of date seeds as promising agents, warranting further extensive research, with the prospect of addressing infections, including gram-positive bacteria^38^. These results were in agreement with those obtained by Barakat et al.^39^ who evaluated an ethanolic seed extract from an Egyptian cultivar, revealing an MIC value of 1.7 mg/ml and 1.2 mg/ml against a strain of *E. coli* O157:H7 and *S. aureus* respectively. Regarding other species such as *L. monocytogenes*, a study carried out by Smaoui et al.^40^ utilized an acetone-ethanol (50:50, v/v) seed extract from a Tunisian cultivar. The study reported MIC values ranging between 0.78 and 3.125 mg/ml and MBC values ranging between 1.56 and 12.48 mg/ml.

It is noteworthy that although several studies have explored alcohol and aqueous extracts of date seeds, to the best of our knowledge, there is notable absence of data regarding the MIC and MBC values of date seed extracts against other foodborne pathogens such as *S. typhimurium* and *S. enteritidis*. Furthermore, studies investigating the antimicrobial efficacy of date seed oil extracts are limited. The findings from current study showed that date seed oil exhibited lower MIC values against certain bacterial strains (10–15 µl/ml), underscoring its higher antimicrobial activity. Essential oils have been reported in previous studies to exhibit higher antimicrobials when compared to their ethanolic and aqueous extract counterparts^41,42^.

Compared to other studies that aimed to identify the phytoconstituents of date seed oil using GC–MS analysis, Qadir et al.^43^ analyzed the hexane fraction of Ajwa date seed oil, revealing 40 peaks. In parallel with our findings, GC–MS analysis of Ajwa date seed oil revealed the presence of several peaks, some of which were identified as hydrocarbons, including two instances of octadecane. However, a notable divergence from our findings was observed in the peak areas, which were significantly lower (0.30% and 1.05%)^43^. This disparity in results could be attributed to various factors including variety, geographic origin, climate, growing conditions, soil type, fertilizer used, cultural methods, storage conditions, different analytical methods used, different standards used, and the solvents used for extraction^44,45^.

The rich hydrocarbon content of date seed oil, their role as antimicrobial agents, and their mechanism of action are of specific interest in this study. The antioxidant activity of hydrocarbons has been reported in various studies on other oils. According to a study carried out by Yassa et al.^46^, the essential oil of the plant *Rosa damascene*, exhibited strong radical scavenging activity, surpassing vitamin E, and butylated hydroxytoluene (BHT). This potency was potentially ascribed to the oil’s rich content of hydrocarbons, including linalool, geraniol, nerol, 1-nanodecene, n-tricosane, n-pentacosane, and n-hexatriacontane, along with other compounds including octadecane. Moreover, variations in the compositions of alkanes present in oils have been used to explain their different inhibitory effects^47^. For instance, in a study assessing the antimicrobial effect of the oils derived from various parts of the Tunisian plant *Allium nigrum* L., including stems, leaves, bulbs, and flowers. It has been shown that these plant organs contain different alkane compositions. While palmitic acid was shown to be the major fatty acid in all organs, bulb oil had the highest percentage of octadecane (30.5%). Notably, bulb oil exhibited inhibitory effects against a range of gram-positive and gram-negative strains, including *St. aureus, Enterococcus faecalis,* and *Proteus mirabilis*, with MIC values of 250, 125, and 250 µl/ml respectively^47^. In a study conducted by Uma and Parvathavarthini^48^, the antibacterial potential of sea urchin extracts against various gram-positive and negative strains, including *Staphylococcus aureus, Bacillus subtilis, E. faecalis, E. coli, Pseudomonas aeruginosa,* and *Klebsiella pneumoniae* was investigated. The hexane extract of the marine plant exhibited a rich composition of alkanes including pentadecane, eicosane, heptadecane, docosane, and heneicosane, which were believed to be responsible for its potent antibacterial effect against all bacterial strains except *Klebsiellapneumoniae*^48^. Likewise, in the current study, date seed oil was shown to constitute various compounds including heptadecane, 1-Nanodecene, n-hexadecanoic acid, tetradecane, pentadecane, heptane, and majorly octadecane which could be used to explain its antimicrobial effect. However, as is the case with other oils, the mechanism of action is still not completely understood^35^. This complexity in determining the precise cause of the antimicrobial effect of oils can be attributed to the interaction of the different classes of active compounds^49^.

The fatty acid composition of date seed oils in our study were found to align with prior research, including an investigation carried out by Golshan Tafti and Panahi^22^. These studies reported that oleic acid and lauric acid constituted approximately 48.1–50.5% and 14–15.8% of these fatty acids, respectively. Additionally, palmitic acid (${\mathrm{C}}_{16:0})$, myristic acid (${\mathrm{C}}_{14:0})$, linoleic acid (${\text{C}}_{18:2}\text{)}$ and stearic acid (${\mathrm{C}}_{18:0})$ were present at percentages ranging from 10.8–11.8%, 10.6–10.9%, 7.7–8.2%, and 3–3.4%, respectively. These results are in agreement with other reported values, such as the work by Laghouiter et al.^50^ where oleic acid levels ranged from 37.83 to 55.0% across nine Algerian cultivars. Furthermore, lauric acid accounted for 6.63–25.36%, myristic acid for 9.30–19.33%, palmitic acid for 9.63–17.59%, linoleic acid for 5.71–10.44%, and stearic acid for 0.77–3.4%. Our results indicate that the oils extracted from the date seeds of Jordaninan Medjoul is also considered an oleic-lauric acid-rich oil^51,52^.

An extensive review of the existing literature indicates that there is a notable scarcity of studies investigating the antimicrobial effect of date seed oil. Only one study, thus far, has investigated this subject, specifically focusing on the lipophilic fraction of three Algerian date seed varieties. This single investigation employed the disc diffusion method to assess the antimicrobial efficacy against different bacteria, including *E. coli, E. faecalis, P. aeruginosa,* and methicillin-resistant *St. aureus* (MRSA). The results from this study showed that the treatment with Deglet Nour and Takerbucht cultivars had highest inhibition zones measuring 19.25 mm and 18.75 mm against *E. faecalis* and *E. coli*, respectively^53^. Our current study is the first to assess the antimicrobial potential of Medjoul date seed oil against different foodborne pathogens, using the broth dilution method. Our research demonstrated that while *St. aureus* was susceptible to inhibition by seed oil, demonstrating an overall reduction of 2.0–6.0 logs, the oil exhibited discrepancies depending on the *St. aureus* strain, with *St. aureus* (ATCC 43300) having a higher sensitivity comparatively and little to no inhibitory effect on *L. monocytogenes* strains. This observation could potentially be explained by the presence of a strain-dependent effect^4^. This lack of significant inhibitory effect of date seed oil against *L. monocytogenes* may also be due to the low range of date seed oil concentrations investigated in this study. Upon further investigation in the literature, we found that other oils such as olive oil had a significant inhibitory effect against *L. monocytogenes* with an MIC value of 1.25 mg/ml^54^. According to the study, olive oil resulted in adenosine 5′-triphosphate (ATP) depletion and further morphological changes including cell membrane damage as evidenced by the leakage of cell fluid^54^. Accordingly, further studies may be required to investigate whether higher date seed oil concentrations would be sufficient to cause significant antimicrobial activities against *L. monocytogenes.* Conversely, in the current study, date seed oil revealed a significant inhibitory effect against the gram-negative bacteria, *E. coli*, *S. enteritidis*, and *S. typhimurium*. This finding is interesting and unanticipated, as gram-positive bacteria are often reported to be more susceptible to bactericidal antimicrobials than gram-negative bacteria^55^. This can be justified by the fact that some antimicrobial agents have a narrow specific activity spectrum, and thus are not active against a particular species or category of microorganisms^56,57^.

In this study, we examined the effect of incubation temperature on the inhibitory potential of date seed oil against various foodborne pathogens. Evidently, date seed oil exhibited a more pronounced inhibitory effects on all foodborne pathogens when incubated at the optimum growth temperature of 37 °C, with the exception of *L. monocytogenes*. This could be attributed to the increased metabolic activity of bacterial cells at optimum growth temperatures^58–60^. At the lower incubation temperature of 10 °C, certain bacterial strains such as *E. coli* O157:H7 (02:0627), *St. aureus* ATCC 33591, and *St. aureus* ATCC 43300 demonstrated similar trends. These strains demonstrated the highest log reductions when exposed to lower concentrations of date seed oil (ranging between 2.5 to 10 µl/ml), potentially due to the saturation of efflux activity in the bacterial cells^61^. Moreover, our results indicated a temperature-dependent effect on the tested foodborne pathogens. Incubation at 10 °C showed a stronger inhibitory effect on the tested gram-negative bacteria than the lower incubation temperature of 4 °C. This observation corroborates the work of Gavriil et al.^50^, who reported similar temperature-dependent outcomes with nine hydro-distilled extracts of various plants including basil, thyme, oregano, rosemary, and other plants. At 37 °C, the plant extracts either reduced or inhibited the growth of three *S. typhimurium* strains, whereas at 4 °C, the plant extracts solely resulted in the reduction of the bacterial cells. This heightened activity can be attributed to the enhanced antibacterial activity with increasing temperatures^58–60^, which could be attributed to the increased active state of the bacterial cells that leads to higher growth and death rates at higher temperatures^62^. Conversely, lower temperatures, decrease the growth rate of the bacterial cells, rendering them less susceptible to the action of the antimicrobial agent^63^. Additionaly, lower temperatures can lower the efficiency of the oils due to the decreased diffusion rates and reduced fluidity of bacterial membranes^64^.

To gain insight into the mechanism of action underlying the observed antimicrobial effects, atomic force microscopy (AFM) analysis was conducted. In terms of the AFM images, *St. aureus* cells exhibited a decrease in length and width following treatment with the date seed oil compared to the control. This change may be attributed to the loss of cytoplasmic content and cell lysis. Conversely, *E. coli* and *Salmonella* spp. cells, showed an increase in length and width, potentially indicative of enhanced permeability of the membrane and cell swelling^65^. Furthermore, we observed a decrease in the average roughness and root mean square roughness in all treated samples, except for *Salmonella* spp. treated with DSO (70 °C/3 h). This reduction in cell roughness has been linked to decreased cell adhesion^66^. Additionally, the formation of blebs, which has been reported in previous studies, is suggested to be due to damage to the membrane components, which consequently leads to the destruction of the cell membrane and wall interaction^67^. Loss of *St. aureus* cluster formation and cell shape irregularity have also been documented in other studies involving essential oils^67,68^. Notably, our findings are consistent with those of previous studies. For instance, Fu et al*.*^28^ investigated the effect of rosemary oil on *Propionibacterium acnes*, and observed binding of the oil to the bacterial cell surface at lower concentrations, along with cell wall desquamation, cytoplasm discharge, and damage to the cell with increasing concentrations. This antimicrobial effect was attributed to the fat-soluble and low-molecular-weight components of the oil, including terpenes and terpenoids^28^. Similarly, Hafedh et al.^69^ also noted a higher susceptibility of gram-negative bacteria as opposed to the gram-positive bacteria when treated with *Mentha longifolia* L. spp essential oil. This susceptibility was linked to the high levels of monoterpenes in the oil, which were thought to be responsible for the disruptions in the outer membrane, such as holes on the cell wall surface, as well as affecting the cytoplasmic membrane or peptidoglycan layer and the release or disentanglement of the exopolysaccharides found in the outer membrane^69^.

Contrary to the common notion that gram-positive bacteria are more susceptible to antimicrobials due to the absence of an outer membrane (OM)^70^, our study indicated greater sensitivity of gram-negative bacteria to date seed oil. This phenomenon may be attributed to the presence of the hydrophilic channels known as porins. These channels are present in the OM and are responsible for regulating the passage of substances through the membrane which depends on the chemical nature of the antimicrobial agent^71^. These channels, typically excluding the passage of hydrophobic molecules; can be weakened by disintegration of the lipopolysaccharide (LPS) layer by molecules known as permeabilizers^72–74^. In contrast, gram-positive bacteria are characterized by a thicker layer of peptidoglycan, rendering the passage of antimicrobials difficult due to the rigidity of the cells, potentially explaining the higher susceptibility of gram-negative bacteria to essential oils^75^. Moreover, it is worth noting that organic solvents at high concentrations are also known to denature proteins by their solubilizing effect on nonpolar side residues by weakening hydrophobic interactions in proteins^76^.

**Figure 6:**
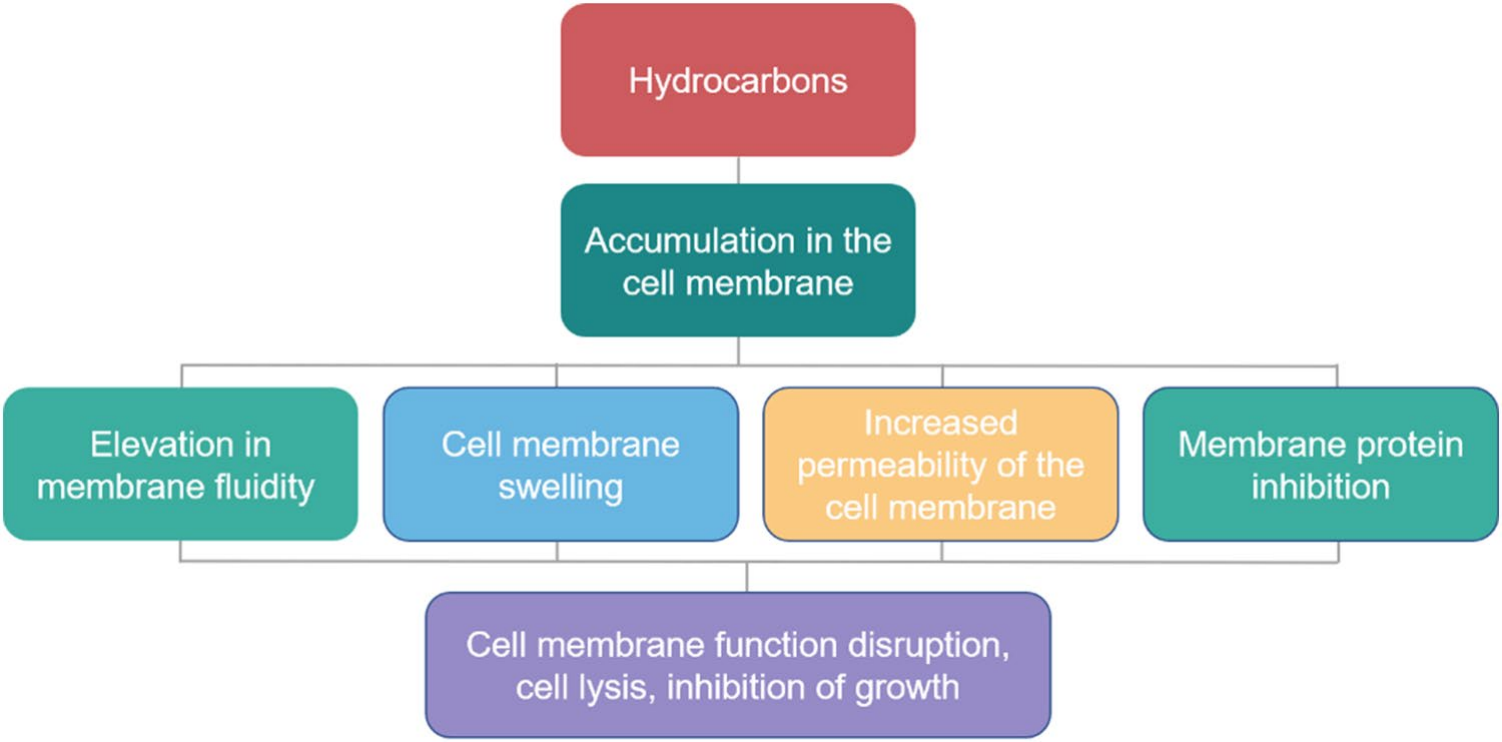
Schematic of the mechanism of action of hydrocarbons on bacterial cell membrane. Adapted from Heipieper and Martínez-Lavanchy^65^.

Our GC–MS analysis revealed that date seed oil comprises a high percentage of saturated and unsaturated aliphatic hydrocarbons, ranging from approximately 66.33% in date seed oil (70 °C/3 h) and 77.75% in date seed oil (50 °C/5 h). The mechanism of action attributed to hydrocarbons is thought to involve their accumulation in the cell membrane, where they interact with the cell membrane or its constituents. This interaction results in the loss of membrane integrity and an increase in the permeability of the membrane to ions and protons^77^. Figure 6 shows a schematic of the mechanism of action of hydrocarbons on cell membranes. Octadecane, the predominant hydrocarbon constituent in date seed oil, comprising approximately half of the constituents, could explain the observed antimicrobial activity. Nonetheless, understanding the precise mechanism of action for oils is complex, and further research is needed to investigate the chiral properties of the present compounds^78^. Furthermore, fatty acid composition analysis of Medjoul date seed oil identified it as oleic-lauric in nature. Because of their aliphatic structure, fatty acids exhibit detergent properties that have been used to explain their detrimental effects on bacterial cells. This property enables them to interact with the cell membrane, forming temporary or permanent pores of varying sizes. At high concentrations, this interaction can result in the release of components from the lipid bilayer, including membrane proteins or larger sections^79,80^. Among saturated fatty acids, lauric acid (C12:0) is considered to have the most potent antimicrobial effect and is reported to exert its antimicrobial effect by means of tubule formation, leading to various defects in the lipid bilayer^55,81^. Oleic acid (C18:1) isolated from the leaves of *Helichrysum pedunculatum*, has also exhibited antimicrobial effects against gram-positive bacterial strains, including *Micrococcus kristinae*, *Staphylococcus aureus*, and *Bacillus subtilis*^82^. In another study, the essential oil of the *Rosa damascene* plant was found to exert a strong antimicrobial effect against the gram-negative bacterium *K. pneumonia*, possibly due to its major compounds including nonadecane, heneicosane, oleic acid, and citronellol (each constituting 24.72%, 19.325%, 17.63%, and 12.61% respectively)^83^. While the precise antimicrobial mechanism of fatty acids remains unclear^55^, the cell membrane is thought to be the first target of oils^84^. Several mechanisms have been proposed, including the ability of oils to degrade the cell wall^85^, damage the cell membrane and membrane proteins^86^, induce the leakage of cellular components as a result of increased permeability, reduce proton motive force, and deplete the ATP pool^87^.

## Conclusion

The chemical analysis of *Phoenix dactylifera* L. seed oil showed that the oil is predominantly an oleic–lauric oil with the dominance of hydrocarbons. Among the identified compounds, octadecane was identified as the major compound comprising 52.22–55.43%. DSO had a significant inhibitory effect, particularly against gram-negative bacteria, both in microbiological media and UHT skim milk. In the case of gram-positive bacteria, DSO showed a significant inhibitory effect against *St. aureus* in microbiological media, although its impact diminished in the food matrix. The effect of the oil against *L. monocytogenes* was considered weak using the investigated oil concentrations. It was demonstrated using AFM imaging that DSO is able to induce considerable alterations in the surface of the bacterial cells suggesting its potential role on the cell membrane. Abnormal cell protrusions and grooves, distortion of distinctive cell shapes and arrangements, as well as cell enlargement in *E. coli* and *Salmonella* spp., and shrinkage in *St. aureus* were evident from the images. Our results highlight the potential of *Phoenix dactylifera* L. seed oil as a potential natural preservative and antimicrobial to address the issue of AMR. The utilization of this by-product, which can be challenging to dispose of opens avenue for further applications in the future including its nanoencapsulation in active food packaging films to preserve various perishable food products.

## Methods

### Date (*Phoenix dactylifera* L.) seed collection and preparation

A permission was obtained from a local Mejdoul date farm located in North Shouneh, Jordan to collect the seeds from their farm. All methods were performed according to the guidelines and regulations at Jordan University of Science and Technology. The obtained seeds were washed thoroughly to remove any remaining flesh from the edible fruit, and they were divided into two treatment groups. The first group was dried at 50 °C for 5 h, whereas the second group of date seeds was dried at 70 °C for 3 h. After drying, the seeds were ground into coarse pieces using a pistol and mortar and then ground using a mechanical grinder (*Mikro-Feinmühle-Culatti,* Germany) into powder with a particle size of 1.5 mm.

### Extraction of date seed oil

Oil extraction of date seeds of both treatments was done using hexane (BBC chemical for lab China) using Soxhlet extraction as reported by Laghouiter et al.^50^. Cellulose thimble (33–100-mm) was filled with date seed powder (25 g). The heating mantle was set to the boiling temperature of the solvent and the process was allowed to run for 6 h. The oils were then weighed after evaporating the solvent using a rotatory evaporator and stored at − 18 °C until further use.

### GC–MS analysis of the phytoconstituents in date seed oil

The phytochemical profile of the oil was analyzed using the method described by Qadir et al.^43^. A gas chromatograph (GC) (SUIMDZU QP2010, Japan) connected with mass spectrometry (MS) with a fused-silica capillary column was used, dimension: 30 m, ID: 0.25 mm, film: 0.25 mm and flow rate of mobile phase carrier gas (Helium) was set at 1.0 ml/min. The oven temperature of the GC instrument was increased from 100 to 260 °C at 10 °C min^−1^, and the injection volume was 5 μl. Samples dissolved in n-hexane and methanol were run in the range of 10–850 m/z, and the results were compared using the Wiley spectral library search program. Mass spectra were detected at 30–35 min.

### Fatty acid composition of date seed oil

The phytochemical profile of the oil was analyzed using the protocol followed by the International Olive Council^88^. Approximately 0.1 g of the oil was added to a 10 ml screw tube and 2 ml heptane was added. The tube was shaken using a vortex mixer, and then 0.2 ml of 2N methanolic potassium hydroxide solution was added and shaken vigorously for 30 s. The mixture was allowed to stratify until the upper layer solution became clear, and the upper layer containing methyl esters was decanted into 2 ml screw vials. The solution was injected into a gas–liquid chromatograph equipped with a flame ionization detector (Column: DB-23 (60 m × 0.25 mm × 0.15 µm)). The injector temperature was maintained at 230 °C, whereas that of the flame ionization detector was maintained at 240 °C. Helium (1.20 ml/min) was used as carrier gas.

### Bacterial strains and culture preparation

The bacterial strains used in this study were *E. coli* O157:H7 (02:0627 and 02:0628) human isolates which were mutated and become nonpathogenic, were obtained from Dr. Rafiq Ahmed, National Microbiology Laboratory, Public Health Agency, Canadian Science Center for Human and Animal Health, Winnipeg, MB, Canada. *S. enteritidis* (CRIFS 1016) and *S. typhimurium* (02:8423) isolated from animal and human sources that have been used in a previous study^89^ were obtained from Dr. Amin Olaimat, Department of Clinical Nutrition and Dietetics, The Hashemite University, Jordan. *L. monocytogenes* (ATCC 7644 and ATCC 19115), and *St. aureus* (ATCC 43300 and ATCC 33591) were obtained from the bacterial collection in the microbiology research laboratory, Department of Nutrition and Food Technology, Jordan University of Science and Technology, Jordan. Each working culture was prepared by streaking the stock bacteria on tryptic soy agar (TSA; Oxoid Ltd., Basingstoke, UK), which was then incubated at 37°C for 24 h to obtain a single colony for subculturing on tryptic soy broth (TSB, Oxoid Ltd., Basingstoke, UK), except for *L. monocytogenes,* in which the TSB was supplemented with 0.6% yeast (Oxoid Ltd, Basingstoke, UK) at 37 °C for 24 h. The final bacterial cell concentration was adjusted to ${10}^{6}$ CFU/ml.

### Antimicrobial activity assay

The antimicrobial activity of date seed oil was assessed using the microdilution method in a 96-well plate according to Wiegand et al.^90^. Briefly, date seed oils from the two treatments (50 °C for 5 h and 70 °C for 3 h) were dissolved in 0.5% dimethyl sulfoxide (DMSO) (Fisher Scientific, UK) to a concentration of 300 µl/ml. Subsequently, a 30 µl/ml solution of the oil in Mueller–Hinton broth (MHB) (Oxoid Ltd. Basingstoke, UK) was prepared as the original stock, and serial dilution was performed to prepare subsequent concentrations 20 µl/ml, 10 µl/ml, 5 µl/ml, 2.5 µl/ml and 1.25 µl/ml. Then, 100 µl of the prepared bacterial suspension for each strain was added to each dilution (100 µl) in a 96-well plate, subsequently, the final concentrations after the addition of the bacterial suspension were 15 µl/ml, 10 µl/ml, 5 µl/ml, 2.5 µl/ml, 1.25 µl/ml, and 0.625 µl/ml. The experiment was repeated three times with replicate determinations under different incubation conditions: 37 °C for 24 h and 10 °C for 7 days. After incubation, the suspension was mixed with a digital pipette and 100 µl of each concentration was plated on TSA and incubated for 24 h at 37 °C for *E. coli, St. aureus, and Salmonella* spp. and 48 h at 37 °C for *L. monocytogenes* to determine the bacterial log reduction.

### Antimicrobial activity of date seed oil in skim ultra-high-temperature (UHT) milk as a food model

The oil concentration that showed the best response in the in vitro test (15 µl/ml) was selected to evaluate its effectiveness against the four foodborne pathogens in commercial skim UHT milk as a food model. All steps were performed under aseptic conditions. A cocktail consisting of two strains of each bacterial culture was inoculated in 10 ml of UHT milk to achieve a final density of 10^5^ CFU/ml. After that, date seed oil was added to the milk to yield a final concentration of 15 µl/ml and the tubes were vortexed for 2 min to achieve good homogeneity. The samples were divided into two groups and stored at two cooling temperatures (4 and 10 °C). The bacterial survival count in log CFU/ml was performed in an appropriate selective medium, xylose lysine deoxycholate agar (XLD, Oxoid Ltd., Basingstoke, UK) for *Salmonella* spp., Baird Parker agar (Oxoid Ltd., Basingstoke, UK) for the *St. aureus* strains, Oxoid Ltd. (Oxoid Ltd., Basingstoke, UK) for the *L. monocytogenes* strains, and sorbitol MacConkey agar (MAC, Oxoid Ltd., Basingstoke, UK) for *E. coli* O157:H7, overlaid with TSA on days 0, 1, 3, 5, and 7. The plates were incubated for 24h at 37°C for *E. coli, St. aureus, and Salmonella* spp. and 48h at 37 °C for *L. monocytogenes*.

### Atomic force microscopy (AFM) imaging

#### Preparation of mica sheets

To prepare the bacterial cells for imaging, mica sheets (Ted Pella Inc., Redding, CA, USA) were used as the mounting surface to immobilize the sample. The mica slides were prepared according to the protocol described by Allison et al.^91^. The mica slides were first cut to an appropriate size that fit into the microscope (approximately 10 × 22 mm). The outer layers of the slides were then cleaved off on both sides using tape. Then, the slides were coated with gelatin by dipping and withdrawing the slides quickly in the warm gelatin solution (0.5 g/100 ml distilled water; 60–70 °C). The slides were then left to dry overnight on a paper towel and supported on an edge under a biosafety cabinet to avoid any contamination.

#### Preparation and mounting of bacterial cells

Bacteria were mounted on gelatin-coated mica. For each of the control and treated bacteria, a cocktail consisting of two strains from each bacterial culture was prepared. For the oil-treated bacteria, 1 ml of each bacterial culture in MHB (OD: 0.08–0.13 at 600 nm) was mixed with 1 ml of the oil treatments at a concentration of 10 µl/ml. The tubes were then incubated at 37 °C for 24 h. After incubation, the tubes were centrifuged (4500 rpm for 10 min) and the pellet was washed with sterilized distilled water. The tubes were centrifuged again, the supernatant was discarded, and the pellet was mixed with 50 µl distilled water and vortexed. From this solution, 20 µl was mounted on a mica slide, left to dry, washed with distilled water, and left to dry. The slides were then prepared for imaging. For the control, bacteria were added to sterilized distilled water (OD: 0.5–1.0 600 nm), followed by the same process. All bacteria in this study were imaged, except for *L. monocytogenes*, as the oil showed no significant effect on bacterial cells. Imaging of the bacterial cells was performed using the AFM non-contact mode (QScan Sync SP mode, AIST-NT), n-type silicon tip (NSC15/Al BS, Mikromasch), nominal spring constant of 40 N/m, resonance frequency of 356 kHz, and scanning rate of 0.3–0.7 Hz. Images were analyzed using the Gwyddion Software.

### Statistical analysis

Data were analyzed using two-way analysis of variance (ANOVA) [IBM® SPSS® Statistics (version 25)]. Data are expressed as mean ± standard error of the mean (SEM). Tukey’s test was performed for pairwise comparisons to identify significant differences between the treatments (p < 0.05).

### Supplementary Information

Supplementary Figure 1.

Supplementary Figure 2.

Supplementary Table S1.

Supplementary Table S2.

Supplementary Table S3.

## Data Availability

All data generated or analyzed during this study are included in this published article (and its Supplementary Information files).
