# Supplementary material for: Date (Phoenix dactylifera L.) seed oil is an agro-industrial waste with biopreservative effects and antimicrobial activity
Source: Sci Rep. 2023 Oct 10;13:17142. doi: 10.1038/s41598-023-44251-y (PMC10564903; doi:10.1038/s41598-023-44251-y)
Supplement: Supplementary file 2 — Supplementary Figure 2. [file 41598_2023_44251_MOESM2_ESM.pdf]

Date (*Phoenix Dactylifera* L.) Seed Oil is An Agro-Industrial Waste with Biopreservative Effects and Antimicrobial Activity

Hana Alkhalidy[1,*], Anas A. Al-Nabulsi[1], Marah Al-Taher[1], Tareq Osaili [1,2], Amin N. Olaimat [3], Dongmin Liu[4]

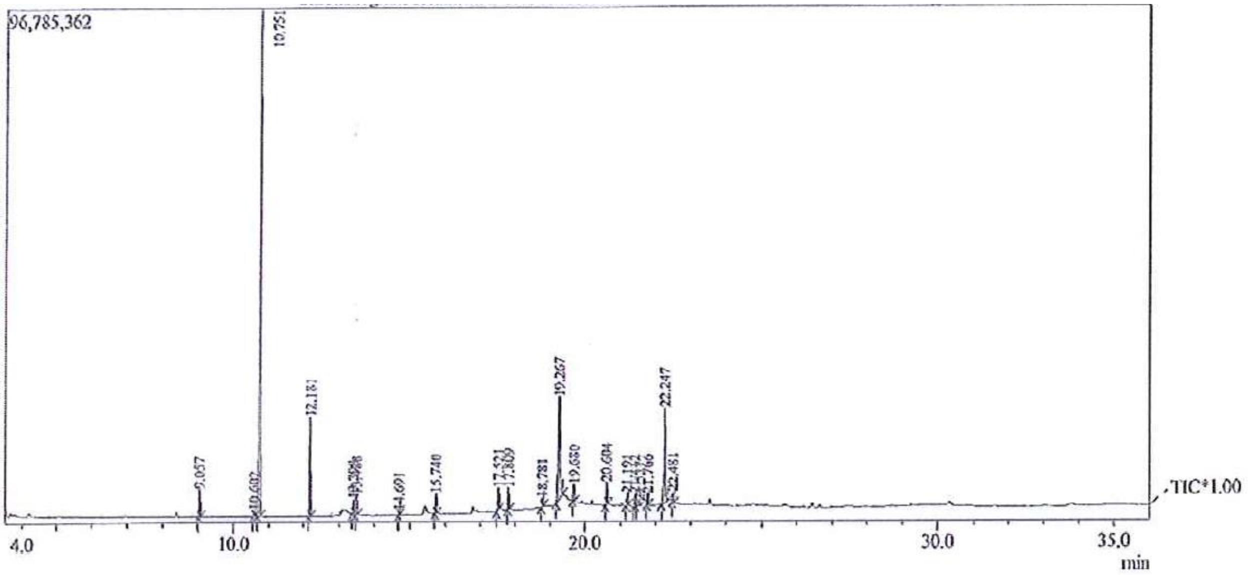

Supplementary Figure 2. GC-MS chromatogram of Medjoul date seed oil (70°C for 3 h)
