# Supplementary material for: Date (Phoenix dactylifera L.) seed oil is an agro-industrial waste with biopreservative effects and antimicrobial activity
Source: Sci Rep. 2023 Oct 10;13:17142. doi: 10.1038/s41598-023-44251-y (PMC10564903; doi:10.1038/s41598-023-44251-y)
Supplement: Supplementary file 3 — Supplementary Table S1. [file 41598_2023_44251_MOESM3_ESM.pdf]

Date (*Phoenix Dactylifera* L.) Seed Oil is An Agro-Industrial Waste with Biopreservative Effects and Antimicrobial Activity

Hana Alkhalidy[1,*], Anas A. Al-Nabulsi[1], Marah Al-Taher[1], Tareq Osaili [1,2], Amin N. Olaimat [3], Dongmin Liu[4]

Supplementary Table S1. GC-MS analysis of Medjoul date seed oil (50°C/5hr).

Peak number	Retention time	Peak area (%)	Compound
1	3.576	8.54	Heptane
2	9.047	2.39	Tetradecane
3	10.748	55.43	Octadecane
4	12.172	6.60	Pentadecane
5	13.387	0.89	1-Heptadecene
6	13.479	1.24	Heptadecane
7	15.730	1.29	1-Nanodecene
8	16.771	1.12	Glycidyl palmitate
9	17.490	1.37	n-Hexadecanoic acid
10	17.626	1.05	4-Estren-4,17 beta-diol-3-one
11	17.795	1.37	1-Nanodecene
12	18.766	0.83	Myristic acid glycidyl ester
13	19.226	5.00	Oleic acid
14	19.665	1.10	n-Tetracosanol-1
15	20.588	1.23	Glycidyl palmitate
16	21.166	0.70	Palmitoleamide
17	21.394	0.81	1-Heptacosanol
18	21.746	0.55	Oleoyl chloride
19	22.123	1.14	Cyclohexane
20	22.227	7.34	9-Octadecenoic acid (Z)
