# Supplementary material for: Date (Phoenix dactylifera L.) seed oil is an agro-industrial waste with biopreservative effects and antimicrobial activity
Source: Sci Rep. 2023 Oct 10;13:17142. doi: 10.1038/s41598-023-44251-y (PMC10564903; doi:10.1038/s41598-023-44251-y)
Supplement: Supplementary file 4 — Supplementary Table S2. [file 41598_2023_44251_MOESM4_ESM.pdf]

Date (*Phoenix Dactylifera* L.) Seed Oil is An Agro-Industrial Waste with Biopreservative Effects and Antimicrobial Activity

Hana Alkhalidy[1,*], Anas A. Al-Nabulsi[1], Marah Al-Taher[1], Tareq Osaili [1,2], Amin N. Olaimat [3], Dongmin Liu[4]

Supplementary Table S2. GC-MS analysis of Medjoul date seed oil (70°C/3hr)

Peak number	Retention time	Peak area (%)	Compound
1	9.057	2.13	Tetradecane
2	10.602	0.43	1-Tetradecene
3	10.751	52.22	Octadecane
4	12.181	6.42	Pentadecane
5	13.398	0.91	1-Heptadecene
6	13.488	1.29	Heptadecane
7	14.691	0.28	Heptadecane
8	15.740	1.30	1-Nanodecene
9	17.521	1.97	n-Hexadecanoic acid
10	17.809	1.35	1-Nanodecene
11	18.781	0.72	Myristic acid glycidyl ester
12	19.267	13.36	Oleic acid
13	19.680	1.08	n-Tetracosanol-1
14	20.604	1.66	Glycidyl palmitate
15	21.191	0.82	Palmitoleamide
16	21.412	0.95	Behenic alcohol
17	21.537	0.48	9-octadecenoic acid
18	21.766	0.81	Oleoyl chloride
19	22.247	10.95	9-Octadecenoic acid (Z)
20	22.481	0.87	Myristic acid glycidyl ester
