# Supplementary material for: Date (Phoenix dactylifera L.) seed oil is an agro-industrial waste with biopreservative effects and antimicrobial activity
Source: Sci Rep. 2023 Oct 10;13:17142. doi: 10.1038/s41598-023-44251-y (PMC10564903; doi:10.1038/s41598-023-44251-y)
Supplement: Supplementary file 5 — Supplementary Table S3. [file 41598_2023_44251_MOESM5_ESM.pdf]

Date (*Phoenix Dactylifera* L.) Seed Oil is An Agro-Industrial Waste with Biopreservative Effects and Antimicrobial Activity

Hana Alkhalidy[1,*], Anas A. Al-Nabulsi[1], Marah Al-Taher[1], Tareq Osaili [1,2], Amin N. Olaimat [3], Dongmin Liu[4]

Supplementary Table S3. Fatty acid composition of Medjoul date seed oil

Fatty acid	Result (%)	
	Date seed oil (50°C/5hr)	Date seed oil (70°C/3hr)
Caproic acid (C6:0)	1.53	1.21
Caprylic acid (C8:0)	0.28	0.31
Capric acid (C10:0)	0.38	0.40
Lauric acid (C12:0)	19.79	20.31
Myristric acid (C14:0)	10.67	10.67
Palmitic acid (C16:0)	9.23	9.11
Palmitoleic acid (C16:1)	0.13	0.12
Margaric acid (C17:0)	0.07	0.07
Margaroleic acid (C17:1)	0.03	0.03
Stearic acid (C18:0)	3.52	3.68
Oleic acid (C18:1)	42.26	43.06
Linoleic acid (C18:2)	9.82	9.78
Linolenic acid (C18:3)	0.22	0.06
Arachidic acid (C20:0)	0.42	0.43
Eicosenoic acid (C20:1)	0.44	0.31
Behenic acid (C22:0)	0.03	0.02
Lignoceric acid (C24:0)	0.05	0.04
